# Influenza-A Viruses in Ducks in Northwestern Minnesota: Fine Scale Spatial and Temporal Variation in Prevalence and Subtype Diversity

**DOI:** 10.1371/journal.pone.0024010

**Published:** 2011-09-13

**Authors:** Benjamin R. Wilcox, Gregory A. Knutsen, James Berdeen, Virginia Goekjian, Rebecca Poulson, Sagar Goyal, Srinand Sreevatsan, Carol Cardona, Roy D. Berghaus, David E. Swayne, Michael J. Yabsley, David E. Stallknecht

**Affiliations:** 1 Southeastern Cooperative Wildlife Disease Study, Department of Population Health, College of Veterinary Medicine, The University of Georgia, Athens, Georgia, United States of America; 2 Daniel B. Warnell School of Forestry and Natural Resources, The University of Georgia, Athens, Georgia, United States of America; 3 Agassiz National Wildlife Refuge, U.S. Fish and Wildlife Service, Middle River, Minnesota, United States of America; 4 Minnesota Department of Natural Resources, Wetland Wildlife Populations and Research Group, Bemidji, Minnesota, United States of America; 5 Department of Veterinary Population Medicine, University of Minnesota, Saint Paul, Minnesota, United States of America; 6 Department of Veterinary Biomedical Sciences, University of Minnesota, Saint Paul, Minnesota, United States of America; 7 Department of Population Health, College of Veterinary Medicine, The University of Georgia, Athens, Georgia, United States of America; 8 Southeast Poultry Research Laboratory, Agricultural Research Service, U.S. Department of Agriculture, Athens, Georgia, United States of America; Erasmus Medical Center, Netherlands

## Abstract

Waterfowl from northwestern Minnesota were sampled by cloacal swabbing for Avian Influenza Virus (AIV) from July – October in 2007 and 2008. AIV was detected in 222 (9.1%) of 2,441 ducks in 2007 and in 438 (17.9%) of 2,452 ducks in 2008. Prevalence of AIV peaked in late summer. We detected 27 AIV subtypes during 2007 and 31 during 2008. Ten hemagglutinin (HA) subtypes were detected each year (i.e., H1, 3–8, and 10–12 during 2007; H1-8, 10 and 11 during 2008). All neuraminidase (NA) subtypes were detected during each year of the study. Subtype diversity varied between years and increased with prevalence into September. Predominant subtypes during 2007 (comprising ≥5% of subtype diversity) included H1N1, H3N6, H3N8, H4N6, H7N3, H10N7, and H11N9. Predominant subtypes during 2008 included H3N6, H3N8, H4N6, H4N8, H6N1, and H10N7. Additionally, within each HA subtype, the same predominant HA/NA subtype combinations were detected each year and included H1N1, H3N8, H4N6, H5N2, H6N1, H7N3, H8N4, H10N7, and H11N9. The H2N3 and H12N5 viruses also predominated within the H2 and H12 subtypes, respectively, but only were detected during a single year (H2 and H12 viruses were not detected during 2007 and 2008, respectively). Mallards were the predominant species sampled (63.7% of the total), and 531 AIV were isolated from this species (80.5% of the total isolates). Mallard data collected during both years adequately described the observed temporal and spatial prevalence from the total sample and also adequately represented subtype diversity. Juvenile mallards also were adequate in describing the temporal and spatial prevalence of AIV as well as subtype diversity.

## Introduction

Wild birds of the order Anseriformes (ducks, geese, and swans) are important reservoirs for avian influenza virus (AIV) [1], [2]. Within these populations, transmission occurs through a fecal-oral route [3]. Prevalence of AIV in ducks varies by species, age, time, and location [2]. Most AIV isolations from North America and Europe have been reported from mallards (*Anas platyrhynchos*) and other species in the subfamily Anatinae, tribe Anatini (i.e., dabbling ducks) [2], [4]–[6]. Juveniles tend to have greater infection rates than adults [1], probably because of the immunologically-naïve status of the former age cohort [4]. In North America, prevalence rates of AIV tend to peak during late summer, when waterfowl aggregate prior to fall migration [1]. Prevalence of infection at that time can be as high as 30% [1]. In contrast, prevalence of AIV infection in ducks on wintering grounds in the southern United States generally is ≤2% [7].

All 16 hemagglutinin (HA) and 9 neuraminidase (NA) subtypes of AIV exist in wild bird populations. However, the H1-12 subtypes predominate in wild duck populations in North America; other subtypes are either associated with gulls (H13, H16) or have not been reported from North America (H14, H15) [1], [5], [6], [8], [9]. Within Anseriform populations in both North America and Europe, the H3, H4, and H6 subtypes consistently account for most of the subtype diversity, and certain HA/NA combinations (e.g. H3N8, H4N6) appear to be over represented in these reported isolates [1], [6], [7], [10], [11].

There have been numerous studies related to the natural history of AIV in wild ducks [5]. Currently, there are global efforts to monitor AIV prevalence in such populations [6], [12]. Although these efforts clearly have defined general spatial, temporal, and species-related epidemiologic patterns at a continental scale, few researchers have examined these relationships on a finer scale. Information related to short-term prevalence or subtype diversity related to fine-scale spatial and temporal variation in sampling efforts not only has application to understanding the epidemiology of these viruses in duck populations, but also the design and implementation of effective and cost-efficient surveillance programs.

In this study, we examined AIV in duck populations in Minnesota, USA from mid-summer to early fall during 2007–2008. The study period corresponds with the pre-migration congregation (hereafter, staging) and southward migration from this geographic area. To provide a historical perspective, we selected study sites at which avian influenza previously had been investigated. At Roseau River Wildlife Management Area (WMA), 7% of 60 mallards sampled during September 1973 were positive for AIV [13]. Similarly, 10.8% of 1,423 mallards and northern pintails (*Anas acuta*) sampled from Marshall and Roseau Counties, Minnesota during September 1998–2000 were positive for AIV [14]. The goals of the current study were to: 1) describe within-season (mid-July to October) variation of AIV prevalence over a small geographic area; 2) determine the spatial, temporal, host, and interacting factors that influence prevalence in our study area; 3) determine the effects of location, time, and host on the detection of subtype diversity; and 4) evaluate sampling efforts necessary to obtain reliable surveillance data from a localized study.

## Methods

### Ethics Statement

All procedures involving animals were approved by the University of Georgia Institutional Animal Care and Use Committee (A2010 6-101). Animals were collected under Minnesota Division of Fish and Wildlife permit 16282 and U.S. Fish and Wildlife Service, U.S. Department of the Interior permit MB779283-0.

### Study Sites

**Figure 1:**
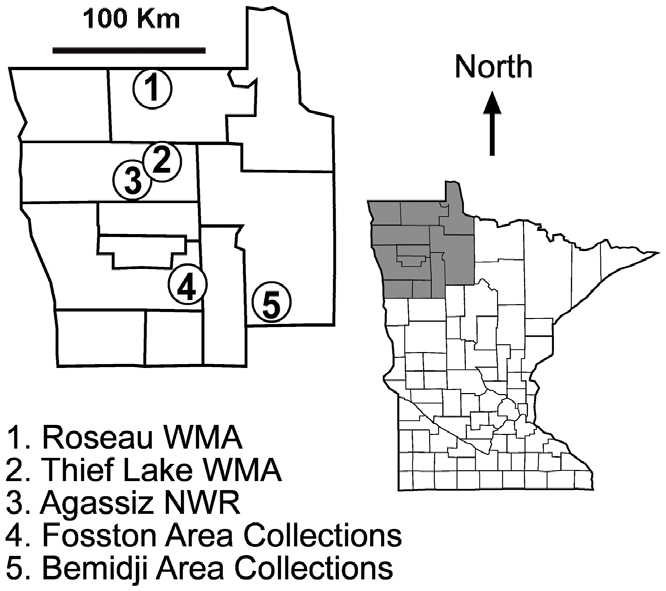
The location of sampled areas for avian influenza virus in northwestern Minnesota, USA, 2007 and 2008.

All study sites were located in Northwestern Minnesota. The study sites were the Bemidji area (Beltrami County - 47°28′25.03″N, 94°52′49.00″W), Fosston area (Polk County - 47°34′36.31″N, 95°45′15.31″W), Thief Lake WMA (Marshall County - 48°29′12.85″N, 95°57′02.17″W), Roseau River WMA (Roseau County - 48°58′39.77″N, 96°00′32.08″W), and Agassiz National Wildlife Refuge (NWR; Marshall County - 48°18′02.90″N, 95°58′49.68″W; Fig. 1).

### Sample Collection

Ducks were captured from July to October of 2007 and 2008 using 3 techniques that were dependent on the time of year: drive trapping, night lighting, and rocket-netting. We also sampled hunter-harvested birds during waterfowl hunting season. Birds were classified as juvenile or adult based on plumage characteristics [15], and AIV samples were collected as previously described [14]. Specifically, cloacal swabs were obtained using sterile cotton-tipped applicators (Puritan® Medical Products Company LLC, Guilford, ME) and placed in 2 ml of Brain Heart Infusion media (Becton Dickinson and Co., Sparks, MD) supplemented with penicillin G (1,000 units/ml), streptomycin (1 mg/ml), kanamycin (0.5 mg/ml), gentamicin (0.25 mg/ml), and amphotericin B (0.025 mg/ml) (Sigma Chemical Company, St. Louis, MO). Samples were stored at 4C (24–72 hrs), shipped overnight, and frozen at −80C until processed.

### Virus Isolation and Subtyping

To isolate viruses, samples were thawed, vortexed for 15 s, and centrifuged at 1,500× *g* for 15 min. The supernatant was inoculated (0.25 ml/egg) into four 9–11 day old specific-pathogen-free (SPF) embryonated chicken eggs via the allantoic route [7]. Eggs were incubated at 37C for 120 hr, after which amnio allantoic fluid was collected and tested by hemagglutination (HA) assay [16]. All HA-positive samples were tested by AIV matrix RT-PCR using primers from Fouchier (2000) [17]. Subtyping was done at the National Veterinary Services Laboratories (NVSL), Ames, Iowa using hemagglutination inhibition and neuraminidase inhibition tests [18] and via genotyping of the HA and NA genes at the University of Minnesota, St. Paul, Minnesota [19], [20] and the University of California Davis [21] using standard Sanger sequencing for HA and NA segments. BLAST non-redundant database searching algorithm was applied to assign specific subtypes.

### Data Analysis

Univariate associations between predictor variables and the results of virus isolation testing were evaluated using a chi-square test of independence. Multivariable analysis was performed using logistic regression with virus isolation as the dependent variable. Robust standard errors were used to account for the lack of independence between birds captured from the same site during a sampling season. Variables having a univariate association (*P*<0.2) with influenza prevalence were eligible for inclusion in the multivariable analysis. Variables were removed from the multivariable model in a stepwise fashion based on their level of significance until only those with *P*<0.1 remained. After reaching a preliminary main-effects model, all possible two-way interactions were evaluated. Date of sample collection was evaluated as both a categorical variable (i.e., month) and as a continuous variable (i.e., week and day), with Akaike's Information Criterion (AIC) being used to determine its most appropriate functional form as a predictor. The fit of the final model was evaluated using the Hosmer-Lemeshow goodness of fit test. Residuals and influence statistics were used to screen for influential covariate patterns by plotting the delta deviance, delta chi-square, and delta beta values versus predicted probabilities. All analyses were performed using commercially available statistical software (Stata version 11.0, StataCorp LP, College Station, TX). Hypothesis tests assumed a two-sided alternative hypothesis and *P*-values <0.05 were considered statistically significant.

## Results

During 2007 (July 11 – October 28) and 2008 (July 10 – October 15), 2,441 and 2,452 ducks were sampled in Minnesota, respectively (Table 1). Avian influenza viruses were isolated from 222 (9.1%) of sampled ducks during 2007 and from 438 (17.9%) of birds during 2008. Based on chi-square test of independence, the prevalence of AIV differed between mallards, other dabbling ducks (Tribe: Anatini), and the combined sample of divers (Tribes: Athyini, Mergini, Oxyurini) and wood ducks (*Aix sponsa*; S1 Table). Peak prevalence in mallards during 2007 and 2008 were approximately 20% and 35%, respectively.

**Table 1:** The prevalence of AIV in waterfowl by species and year from northwestern Minnesota, USA.

Species^a^	2007		2008		Total	
	N	No. Pos (%)	N	No. Pos (%)	N	No. Pos (%)
Mallard (*Anas platyrhynchos*)	1691	185 (11.0%)	1426	346 (24.3%)	3117	532 (17.1%)
Other Dabblers						
Gadwall (*Anas strepera*)	12	1 (8.3%)	13	1 (7.7%)	25	2 (8.0%)
American Wigeon (*Anas americana*)	10	1 (10.0%)	52	3 (5.8%)	62	4 (6.5%)
American Black Duck(*Anas rubripes*)	8	1 (12.5%)	1	0 (0%)	9	1 (11.1%)
Blue-winged Teal (*Anas discors*)	193	21 (10.9%)	468	31 (6.6%)	661	52 (7.9%)
Northern Shoveler (*Anas clypeata*)	10	0 (0%)	61	26 (42.6%)	71	26 (36.6%)
Northern Pintail (*Anas acuta*)	31	2 (6.5%)	47	6 (12.8%)	78	8 (10.3%)
Green-winged Teal (*Anas crecca*)	116	5 (4.3%)	191	23 (12.0%)	307	28 (9.1%)
Wood Ducks and Divers						
Wood Duck (*Aix sponsa*)	186	0 (0%)	20	1 (5%)	206	1 (0.5%)
Redhead (*Aythya americana*)	3	1 (33.3%)	12	0 (0%)	15	1 (6.7%)
Ring-necked Duck (*Aythya collaris*)	83	4 (4.8%)	138	1 (0.7%)	221	5 (2.3%)

a Also includes Mallard/Gadwall Hybrid (n = 1, 2007, *Anas platyrhynchos/strepera*); Mallard/American Black Duck Hybrid (n = 5, 2007; 1, 2008, *Anas platyrhynchos/rubripes*); Canvasback (n = 18, 2007; 1, 2008, *Aythya valisineria*); Greater Scaup (n = 2, 2007, *Aythya marila*); Lesser Scaup (n = 23, 2007; 11, 2008, *Aythya affinis*); Bufflehead (n = 5, 2007; 3, 2008, *Bucephala albeola*), Common Goldeneye (n = 24, 2007; 6, 2008, *Bucephala clangula*); Hooded Merganser (n = 3, 2007, *Lophodytes cucullatus*); Common Merganser (n = 17, 2007, *Mergus merganser*); and Ruddy Duck (n = 1, 2008, *Oxyura jamaicensis*). AIV was not isolated from any of these species.

**Figure 2:**
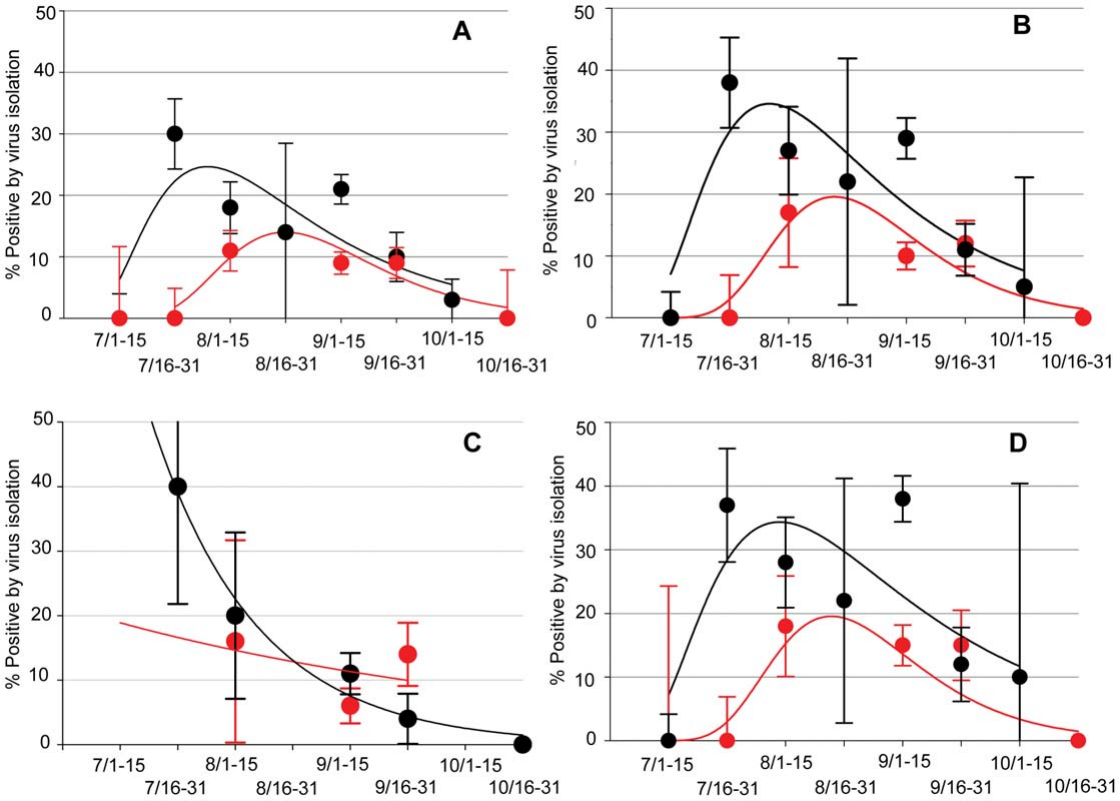
Epidemic curves showing the within-year temporal change in percent of ducks in northwestern Minnesota infected with avian influenza virus, 2007 (red lines) and 2008 (black lines; A =  all species; B =  Mallards; C =  Adult Mallards; and D =  Juvenile Mallards). Trend lines were calculated using SigmaPlot software (Systat Software Inc., Richmond California, USA) using a peak, log normal, 3 parameter function (A.B, and D) and exponential decay, single, 2 parameter functions (C).

During both 2007 and 2008 AIV prevalence peaked in late summer (late August or early September; Fig. 2). Based on chi-square testing of independence, prevalence significantly differed between years, month of sampling, age, and location (Table S1). Because the characteristics of sampled birds varied over time and location, a multivariable logistic regression analysis was performed to evaluate potential interactions and obtain adjusted effect estimates. The low prevalence of AIV in both the Fosston and Bemidji locations resulted in computational problems when interactions between location and the other variables were evaluated, and consequently observations from Fosston and Bemidji (*n* = 330) were excluded from the multivariable analysis. Because the preliminary analysis identified interactions between year, sampling date, and location; intermediate model selection steps were performed separately for each year. Ultimately, the same combination of predictors was identified as the best fitting model based on AIC comparisons for both 2007 and 2008. Both of these year-specific models (not reported) included an interaction between location and sampling date. The best fit of both models occurred when sampling date was included as the week of sample collection in its quadratic form (i.e., week and week^2^). The sampling week variable was centered by subtracting the mean (week 36) from each observation to prevent collinearity with the squared week term. Because models for both years shared the same predictors, data from 2007 and 2008 were combined in the final multivariable model, which contained a significant three-way interaction between location, week of sample collection, and year. All interactions and main effects in the final model were also statistically significant, and results of the Hosmer-Lemeshow test indicated that the model fit the data well (H-L goodness-of-fit test, *P* = 0.84).

**Figure 3:**
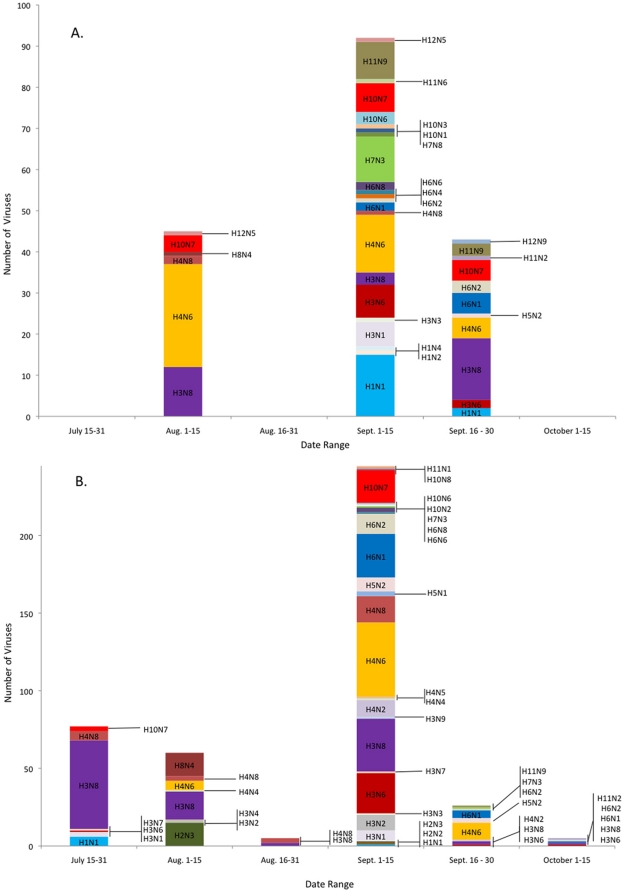
The temporal variation of subtype diversity of avian influenza viruses from ducks captured in northwestern Minnesota, USA (A = 2007; B = 2008). This graph demonstrates that the peak collection period to gather subtypes is early September.

Results of the final multivariable model are reported in Table 2. Adjusted effect estimates for the age and species type variables in this model indicated that the odds of having a positive virus isolation result were 2.7 times higher for juvenile birds than for adults, and compared to mallards, odds ratios were 0.10 for divers and wood ducks, and 0.32 for the other dabblers. Overall, subtypes were determined for 607 of the 660 (93.5%) AIV isolated. During 2007, 27 subtypes were detected in 190 AIV isolates, and 31 subtypes were detected in 417 AIV isolates during 2008 (Fig. 3). Predominant subtypes during 2007 (representing at least 5% of subtype diversity) were H1N1, H3N6, H3N8, H4N6, H7N3, H10N7, and H11N9. Predominant subtypes during 2008 were H3N6, H3N8, H4N6, H4N8, H6N1, and H10N7. Overall, 10 HA subtypes were detected each year: H1, 3–8, and H10–12 during 2007 and H1–8, 10 and 11 during 2008. All NA subtypes were represented each year.

**Table 2:** Multivariable logistic regression model for the prediction of avian influenza virus isolation results in 4,563 waterfowl sampled in northwestern Minnesota, USA, 2007 and 2008.

Variable	Coefficient	†Robust SE	Odds Ratio (95% CI)	‡P
**Year**				
2007 (1)	Referent		NC*	<0.001
2008 (2)	1.658	0.079		
**Location**				
Thief Lake WMA (1)	Referent		NC	<0.001
Roseau River WMA (2)	−0.055	0.145		
Agassiz NWR (3)	−2.270	0.101		
**Week (centered)**	1.163	0.043	NC	<0.001
**(Week)^2^**	−0.392	0.006	NC	<0.001
**Age**				
Adult	Referent			<0.001
Juvenile	0.996	0.181	2.7 (1.4, 3.9)	
**Species type**				
Mallard	Referent			<0.001
Other Dabblers	−1.147	0.142	0.32 (0.24, 0.42)	
Divers & Wood Ducks	−2.313	0.407	0.10 (0.04, 0.22)	
**Location X Week**				
2	−1.424	0.097	NC	<0.001
3	−0.362	0.101		
**Location X (Week)^2^**				
2	0.376	0.009	NC	<0.001
3	0.402	0.011		
**Year X Location**				
2 2	−0.854	0.191	NC	<0.001
2 3	1.416	0.197		
**Year X Week**				
2	−2.892	0.055	NC	<0.001
**Year X Location X Week**				
2 2	3.078	0.102	NC	<0.001
2 3	1.810	0.126		
**Constant**	−2.674	0.160	---	<0.001

† Robust standard error (SE) adjusted for clustering within capture sites (*n* = 24).

‡ P-value based on Wald chi-square statistics.

*NC – Not calculated because the odds ratio depends on the level of the interacting variables.

Although subtype diversity varied between years (Fig. 3), similar patterns were observed during each year. During both years, the diversity of subtype combinations increased with the total number of isolates (Figure S1). During 2007, six subtypes were detected in viruses isolated during August; this increased to 23 subtype combinations isolated during early September and decreased to 11 subtypes detected during late September. During 2008, 13 subtypes were represented in July/August isolations, 27 subtypes in early September isolates, and 11 were detected during late September/early October. An overlap between predominant subtype combinations was observed between years with the H3N6, H3N8, H4N6, and H10N7 viruses, each comprising more than 5% of total isolates each year (Table 3). Additionally, within each HA subtype, the same predominant HA/NA subtype combinations were detected each year and included H1N1, H3N8, H4N6, H5N2, H6N1, H7N3, H8N4, H10N7, and H11N9. The H2N3 and H12N5 viruses also predominated within the H2 and H12 subtypes, respectively, but were only represented in a single year (H2 and H12 viruses were not detected during 2007 and 2008, respectively).

**Table 3:** Predominant hemagglutinin and neuraminidase subtype combinations detected in northwestern Minnesota, USA, 2007 and 2008, with a comparison to previous studies in North America and Europe.

Subtypes detected and percent (%)					
Alberta 1976–1990(Sharp et al., 1997)	Alberta 1976–2001(Krauss et al., 2004)	Northern Europe 1998–2007(Munster et al., 2007)	2007 Minnesota	2008 Minnesota	Total Minnesota
H1N1 (2.6)	H1N1 (2.1)	H1N1 (6.0)	H1N1 (8.1)	H1N1 (1.6)	H1N1 (3.8)
H2N3 (0.7)	H2N3 (0.6)	H2N3 (4.2)	ND†	H2N3 (3.4)	H2N3 (2.3)
H3N8 (23.3)	H3N8 (22.8)	H3N8 (6.3)	H3N8 (15.3)	H3N8 (26.0)	H3N8 (22.4)
H4N6 (14.6)	H4N6 (12.5)	H4N6 (16.0)	H4N6 (20.7)	H4N6 (14.8)	H4N6 (16.8)
H5N2 (0.2)	H5N2 (0.2)	H5N2 (3.0)	H5N2 (0.5)	H5N2 (2.7)	H5N2 (2.0)
H6N2 (26.3)	H6N2 (20.8)	H6N2 (9.9)	H6N1 (3.2)	H6N1 (7.8)	H6N1 (6.2)
H7N3 (0.6)	H7N3 (0.7)	H7N7 (10.5)	H7N3 (5.4)	H7N3 (0.5)	H7N3 (2.1)
H8N4 (0.4)	H8N4 (0.3)	H8N4 (1.8)	H8N4 (0.5)	H8N4 (3.4)	H8N4 (2.4)
H9N1 (0.07)	H9N1 (0.1)	H9N2 (1.2)	ND	ND	ND
H10N7 (0.6)	H10N1, H10N6 (0.09)	H10N7 (1.2)	H10N7 (7.7)	H10N7 (5.5)	H10N7 (6.2)
H11N9 (0.6)	H11N9 (0.08)	H11N9 (4.8)	H11N9 (5.9)	H11N9 (0.2)	H11N9 (2.1)
H12N5 (0.3)	H12N5 (0.5)	H12N5 (1.2)	H12N5 (0.9)	ND	H12N5 (0.3)

† ND – Subtype not detected.

**Figure 4:**
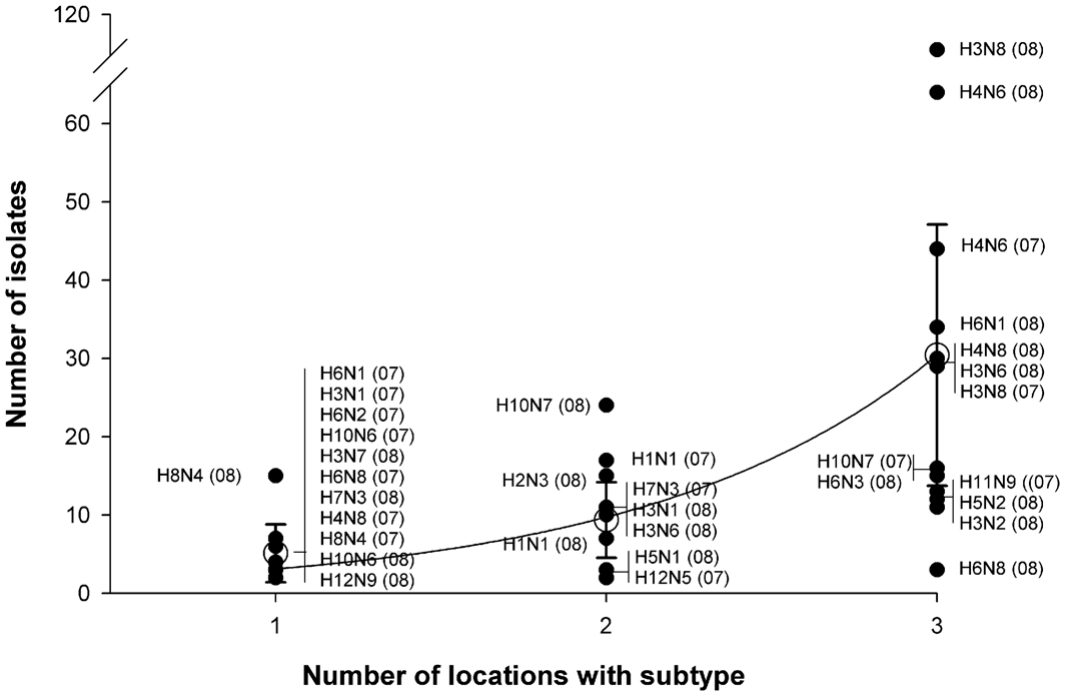
The number of subtype combinations of avian influenza virus that were detected in ducks captured at multiple sites in northwestern Minnesota, USA, 2007 and 2008.

Subtype diversity detected at the three northwestern study sites (Thief Lake WMA, Roseau River WMA, and Agassiz NWR) is reported in Table 4. Only subtypes represented by more than one isolate during 2007 or 2008 are included and overall these included 13 and 20 subtypes during those years, respectively (Table 4). For the combined years, 25 of the 33 subtypes (76%) were detected at more than one of the study sites. The probability of detection of a specific subtype at multiple locations increased with the number of isolates of that subtype (Fig. 4).

**Table 4:** Spatial variation in AIV subtypes from sites sampled in northwestern Minnesota, USA, 2007 and 2008.

	2007				2008			
Subtypes	Thief Lake WMA	Roseau River WMA	Agassiz NWR	n	Thief Lake WMA	Roseau River WMA	Agassiz NWR	n
H1N1	15	2		17	4		3	7
H2N2				0		1	1	2
H2N3				0	1	14		15
H3N1	6			6	6		4	10
H3N2				0	6	3	2	11
H3N6	6		4	10	11	9	9	29
H3N7				0			2	2
H3N8	1	16	12	29	20	28	66	114
H4N2				0	3	3	6	12
H4N4					1	1		2
H4N6	13	28	3	44	27	18	19	64
H4N8		1		1	14	5	11	30
H5N1				0	1	2		3
H5N2		1		1	3	2	7	12
H6N1		7		7	4	18	12	34
H6N2		4		4	2	7	6	15
H6N8		2		2	1	1	1	3
H7N3	10		1	11			2	2
H8N4		1		1		15		15
H10N6	3			3	1			1
H10N7	6	6	4	16	3		21	24
H11N9	9	2	2	13			1	1
H12N5	1	1		2				
Total	70	71	26	167	108	127	173	408

Mallards were the predominant species sampled (63.7% of the total sample) and were the species with the greatest prevalence of AIV (80.5% of total detections). To determine whether surveillance of this species would adequately describe temporal variations of prevalence and subtype diversity at the northwestern Minnesota study sites, we compared annual data derived from mallards and from the total sample (i.e., all species). The mallard data in both years adequately described temporal prevalence (Fig. 2) and subtype diversity (Table S2). With regard to subtype diversity, only one subtype combination detected in the 2007 total sample and four 2008 subtype combinations were not represented in mallards during those years. These were an H6N6 detected in a northern pintail sampled during 2007 and an H3N9 from a green-winged teal (*Anas crecca*), H4N5 from a green-winged teal, H10N6 from a blue-winged teal (*Anas discors*), and H10N8 from an American wigeon (*Anas Americana*) that were isolated during 2008. Juvenile mallards comprised 39.6% of the total sample. Further, all but four detected subtypes (H1N4, H6N6, H7N8, and H8N4) were represented in juvenile mallards in 2007, and only five of the total subtypes (H3N9, H4N5, H10N6, H10N8 and H11N9) were not isolated from juvenile mallards detected from 2008 samples. Overall, the temporal pattern of AIV in juvenile mallards (Fig. 2D) was similar to that observed for the total duck sample (Fig. 2A) and the entire mallard sample (Fig. 2B) during both years.

## Discussion

Our results indicate a seasonal trend in AIV prevalence within northwestern Minnesota that is consistent with previously described seasonal patterns in North America. This trend consists of a seasonal peak that starts in late July and peaks during August [2], [4]. Locally, our observations also are consistent with a previous study in Minnesota from 1980–1983 that used a sentinel mallard system. Specifically, infected mallards in the earlier study first were detected during late July and early August during all four years of that study [22]. Prevalence and the specific timing of this peak varied slightly between years (Fig 2). Further, our predictive multivariable model suggests that temporal patterns may not be synchronized among study sites (Figure S1). Variations in seasonality were apparent in previous studies conducted in North America [23] and Europe [12]. In New York, prevalence peaked in late September [23], and in Europe, the peak appeared to extend from September until December [12]. These differences may be influenced by variations of migratory timing, population structure, or species abundance and diversity at different sites.

Consistent with previous studies [2], [4], [6], AIV prevalence was dependent on species and age. Mallards were much more likely to be infected than the combined samples of dabbling ducks or the diving ducks and wood ducks. Overall, prevalence in the dabbling duck species included in this study ranged from 7% in American wigeon to 37% in northern shoveler (*Anas clypeata*). These species-specific differences may be attributed to population density (mallards), behavior (dabbling ducks and wood ducks), feeding habits (surface feeding) [24], or possibly differences in susceptibility (diving ducks and wood ducks). Mallards have been suggested as the most important duck species related to AIV natural history, but the potential roles of other species in the maintenance and long-range movement of AIV should not be discounted. For example, a high prevalence of AIV infection in northern shoveler has been reported from birds sampled on waterfowl wintering grounds [2]. Likewise, both blue-winged teal and northern pintail have been suggested in the long-distance movement of these viruses [7], [25].

Juveniles were almost three times more likely to be infected with AIV than adults (Table 2). This relationship has been previously reported [4] and has been observed in numerous studies worldwide [5], [6], [12]. This may be related to pre-migration staging and the resultant concentration of immunologically naïve juveniles [4].

Because our sampling strategy identified interactive effects between location and time (2 week sampling periods) (Table 2), the marginal effects of location on AIV prevalence could not be independently evaluated. Because environmental and habitat-related characteristics of a wetland (e.g., water depth, water temperature and pH, or avian species composition utilizing the habitat) potentially could influence AIV transmission rates [26], additional work is needed to fully understand this potentially important variable.

Subtype diversity varied over the study period during both years, but most North American HA and NA subtypes were detected during both 2007 and 2008 (Fig. 3). Subtype diversity reflected the observed AIV prevalence (Fig. 2). Few virus subtypes were found early (early July) in either field season, but the number of subtypes increased from August into September and decreased in late September and October. The increased subtype diversity over time may reflect increased detectability related to peak prevalence, the introduction of new subtypes related to migration into the study sites, or reassortment events. In relation to detectability, subtype-specific prevalence (Table S2) often is very low. Specifically, the prevalence of an individual subtype (all species) ranged from 0.04–1.88% and 0.04–4.65% during 2007 and 2008, respectively. Two observations about migration were consistent between years. During both years, several predominant subtypes detected in July and August persisted to late September/early October (H3N8, H4N6, and H10N7 during 2007; H3N8 and H4N6 during 2008). Additionally, several subtypes (H7N3, H11N9, H1N1 during 2007 and H6N1 during 2008) were not detected until early September, when migrating birds probably began arriving in northwestern Minnesota.

The significance of reassortment to the natural history of AIV in wild bird populations is not well defined. The diversity observed within the H3 and H4 subtypes during 2007 and 2008 suggests that reassortment is common. During both years, H3N8 and H4N6 were the predominant subtypes. Numerous NA subtypes were associated with these HA types (H3N1, H3N3, H3N6, H4N8 during 2007; H3N1, H3N2, H3N3, H3N4, H3N6, H3N7, H3N9, H4N2, H4N4, H4N5, H4N8 during 2008). Similar diversity was associated with H6, H10, and H11 viruses during 2007 and H6 and H10 viruses during 2008 (Table S2). In contrast, only one subtype combination was observed with the H8 subtype (H8N4): this cannot be explained but is consistent with other studies (Table S2). Reassortment events are dependent on AIV coinfections, which have been reported in wild duck populations [10]. Because it is intuitive that the likelihood of coinfection is dependent on the prevalence of the potential parent viruses, the relationship between subtype diversity within an HA subtype and the number of isolates recovered is predictable (Fig. 4).

Although the subtypes detected varied annually, most HAs were detected each year. The dominant HA subtypes reported in North American waterfowl are H3, H4, and H6 viruses [2], which may cycle in these populations because of temporal changes in flock immunity [11]. Other HA subtypes have been reported as underrepresented (H2, H5, H7, H8, H9, and H13) or occur sporadically in ducks (H1, H10, H11, and H12) [11]. In our study, HA diversity was consistent with this pattern. The H4 viruses were most common during 2007, the H3 viruses were most common during 2008, and the H6 viruses were well represented during 2008. With the exception of H2, H9 and H12, all other HA subtypes were represented both years but prevalence often was very low (Table S2). The detection of most of these subtypes each year appears to reflect sampling intensity and the high number of isolates recovered. This observation implies that almost all of these HA subtypes are present at the northwestern Minnesota study sites annually.

During both 2007 and 2008, some subtype combinations were over represented. The predominant subtype combinations within the HA types were H1N1, H2N3, H3N8, H4N6, H5N2, H6N1, H7N3, H8N4, H10N7, H11N9, and H12N5. This was consistent between years. Interestingly, with the exception of H6N1 and H7N3 (H7N7 in Europe) [6], these are the same subtype combinations that have been most reported from ducks in long-term studies in North America [2], [4], [23] and Europe [6], [12] (Table 3). It is unknown why these predominant subtypes have been consistently detected in North America since 1976.

Our results suggest that AIV surveillance in northwestern Minnesota could be efficiently achieved by limiting testing to juvenile mallards (Fig. 2; Table S2). Prevalence and subtype diversity could be adequately described with a much reduced sample size based on juvenile mallards alone: in 2007, juvenile mallards represented 35.7% (871/2441) of the total sample and in 2008 juvenile mallards represented 47.3% (1,065/2252) of the total ducks sampled. These results likely were influenced by the large proportion of mallards (Table S1) in our total sample, but this approach should be considered when testing avian communities that are dominated by this species. Such an approach has application to efficiently recovering AIVs from the field and detecting annual variations in prevalence. This approach may not be applicable to situations in which the ecology of these viruses is less understood, where study questions relate the role of individual species in the natural history of these viruses, where mallards are not the predominant species, or in studies on the wintering grounds of North American waterfowl. Additionally, some subtypes such as the H9, H13, and H16 viruses are underrepresented or absent in ducks and may not be detected by this approach. Finally, although subtype diversity was captured, additional work is needed to determine if genetic diversity was adequately represented in the viruses recovered.

## Supporting Information

Figure S1 **The number of subtype combinations associated with each HA type and the number of those HA subtype detected during 2007 and 2008 (• = 2007 and ○ = 2008).** The line is defined by the quadratic function *f = *9.4889−3.9055*x+*2.9441*x^2^* (*r^2^ = 0.7607*, *P<0.0001*).
(TIF)

Table S1 **Summary of avian influenza virus isolation testing results for waterfowl sampled in northwestern Minnesota, USA, 2007 and 2008.**
(DOCX)

Table S2 **Subtype combination found in all sampled duck species, all mallards, and juvenile mallards and their associated rate of recovery from the total number of birds sampled in northwestern Minnesota, USA, 2007 and 2008.**
(DOCX)
