# Supplementary figures and images for: Influenza-A Viruses in Ducks in Northwestern Minnesota: Fine Scale Spatial and Temporal Variation in Prevalence and Subtype Diversity
Source: PLoS One. 2011 Sep 13;6(9):e24010. doi: 10.1371/journal.pone.0024010 (PMC3172203; doi:10.1371/journal.pone.0024010)

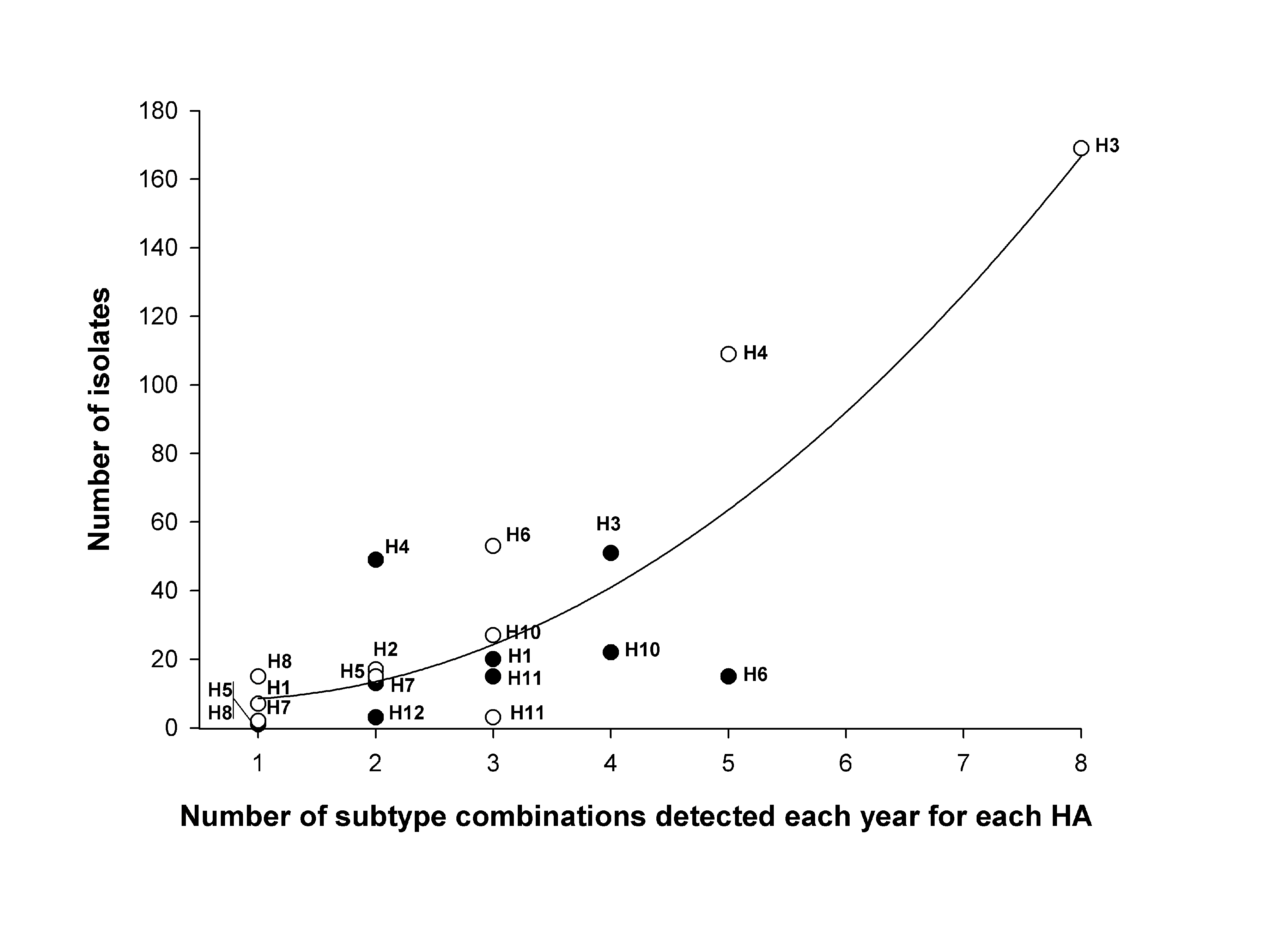

Supplement: Figure S1 — The number of subtype combinations associated with each HA type and the number of those HA subtype detected during 2007 and 2008 (• = 2007 and ○ = 2008). The line is defined by the quadratic function f = 9.4889−3.9055x+2.9441x2 (r2 = 0.7607, P<0.0001). (TIF) [file pone.0024010.s001.tif]
