# Supplementary material for: Influenza-A Viruses in Ducks in Northwestern Minnesota: Fine Scale Spatial and Temporal Variation in Prevalence and Subtype Diversity
Source: PLoS One. 2011 Sep 13;6(9):e24010. doi: 10.1371/journal.pone.0024010 (PMC3172203; doi:10.1371/journal.pone.0024010)
Supplement: Table S1 — Summary of avian influenza virus isolation testing results for waterfowl sampled in northwestern Minnesota, USA, 2007 and 2008. (DOCX) [file pone.0024010.s002.docx]

| Variable | Sample Size | No. Positive (%) | ^†^P |
| --- | --- | --- | --- |
| Year  2007  2008 | 2,441  2,452 | 222 (9.1)  438 (17.9) | < 0.001 |
| Month  July  August  September  October | 471  832  3,345  245 | 82 (17.4)  118 (14.2)  454 (13.6)  6 (2.5) | < 0.001 |
| Species Type  Mallards  Other Dabblers  Divers & Wood Ducks | 3,117  1,221  555 | 531 (17.1)  120 (9.8)  7 (1.3) | < 0.001 |
| Age  Juvenile  Adult | 3,170  1,723 | 543 (17.1)  117 (6.8) | < 0.001 |
| Location  Thief Lake WMA  Roseau River WMA  Agassiz NWR  Fosston / Bemidji | 1,036  1,820  1,707  330 | 209 (20.2)  216 (11.9)  232 (13.6)  3 (0.9) | < 0.001 |
| Total | 4,893 | 660 (13.5) |  |
| ^†^Chi-square test of independence | | | |
