# Supplementary material for: Influenza-A Viruses in Ducks in Northwestern Minnesota: Fine Scale Spatial and Temporal Variation in Prevalence and Subtype Diversity
Source: PLoS One. 2011 Sep 13;6(9):e24010. doi: 10.1371/journal.pone.0024010 (PMC3172203; doi:10.1371/journal.pone.0024010)
Supplement: Table S2 — Subtype combination found in all sampled duck species, all mallards, and juvenile mallards and their associated rate of recovery from the total number of birds sampled in northwestern Minnesota, USA, 2007 and 2008. (DOCX) [file pone.0024010.s003.docx]

| **2007** | | | | | | **2008** | | | | | |  |
| --- | --- | --- | --- | --- | --- | --- | --- | --- | --- | --- | --- | --- |
| **Subtypes** | **All Species** | **Percent Prevalence** | **Mallards** | **Percent Prevalence** | **Juvenile Mallards** | **Percent Prevalence** | **All Species** | **Percent Prevalence** | **Mallards** | **Percent Prevalence** | **Juvenile Mallards** | **Percent Prevalence** |
| H1N1 | 18 | 0.74 | 17 | 1.00 | 11 | 1.26 | 7 | 0.29 | 4 | 0.28 | 3 | 0.28 |
| H1N2 | 1 | 0.04 | 1 | 0.06 | 1 | 0.11 |  |  |  |  |  |  |
| H1N4 | 1 | 0.14 | 1 | 0.06 |  |  |  |  |  |  |  |  |
| H2N2 |  |  |  |  |  |  | 2 | 0.08 | 2 | 0.14 | 2 | 0.19 |
| H2N3 |  |  |  |  |  |  | 15 | 0.61 | 15 | 1.05 | 13 | 1.22 |
| H3N1 | 6 | 0.24 | 6 | 0.35 | 3 | 0.34 | 10 | 0.41 | 8 | 0.56 | 5 | 0.47 |
| H3N2 |  |  |  |  |  |  | 11 | 0.45 | 8 | 0.56 | 8 | 0.75 |
| H3N3 | 1 | 0.04 | 1 | 0.06 | 1 | 0.11 | 1 | 0.04 | 1 | 0.07 | 1 | 0.09 |
| H3N4 |  |  |  |  |  |  | 1 | 0.04 | 1 | 0.07 | 1 | 0.09 |
| H3N6 | 10 | 0.40 | 10 | 0.59 | 7 | 0.80 | 29 | 1.18 | 22 | 1.54 | 19 | 1.78 |
| H3N7 |  |  |  |  |  |  | 2 | 0.08 | 2 | 0.14 | 2 | 0.19 |
| H3N8 | 34 | 1.39 | 28 | 1.66 | 11 | 1.26 | 114 | 4.65 | 76 | 5.33 | 69 | 6.48 |
| H3N9 |  |  |  |  |  |  | 1 | 0.04 |  |  |  |  |
| H4N2 |  |  |  |  |  |  | 12 | 0.49 | 9 | 0.63 | 8 | 0.75 |
| H4N4 |  |  |  |  |  |  | 2 | 0.08 | 1 | 0.07 | 1 | 0.09 |
| H4N5 |  |  |  |  |  |  | 1 | 0.04 |  |  |  |  |
| H4N6 | 46 | 1.88 | 24 | 1.42 | 17 | 1.95 | 65 | 2.65 | 53 | 3.72 | 45 | 4.23 |
| H4N8 | 3 | 0.12 | 3 | 0.18 | 2 | 0.23 | 29 | 1.18 | 25 | 1.75 | 24 | 2.25 |
| H5N1 |  |  |  |  |  |  | 3 | 0.12 | 2 | 0.14 | 1 | 0.09 |
| H5N2 | 1 | 0.04 | 1 | 0.06 | 1 | 0.11 | 12 | 0.49 | 11 | 0.77 | 11 | 1.03 |
| H6N1 | 7 | 0.29 | 7 | 0.41 | 7 | 0.80 | 34 | 1.39 | 28 | 1.96 | 26 | 2.44 |
| H6N2 | 4 | 0.16 | 2 | 0.12 | 2 | 0.23 | 15 | 0.61 | 14 | 0.98 | 13 | 1.22 |
| H6N4 | 1 | 0.04 | 1 | 0.06 | 1 | 0.11 |  |  |  |  |  |  |
| H6N6 | 1 | 0.04 |  |  |  |  | 1 | 0.04 | 1 | 0.07 | 1 | 0.09 |
| H6N8 | 2 | 0.08 | 2 | 0.12 | 1 | 0.11 | 3 | 0.12 | 3 | 0.21 | 3 | 0.28 |
| H7N3 | 12 | 0.49 | 12 | 0.71 | 7 | 0.80 | 2 | 0.08 | 2 | 0.14 | 1 | 0.09 |
| H7N8 | 1 | 0.04 | 1 | 0.06 |  |  |  |  |  |  |  |  |
| H8N4 | 1 | 0.04 | 1 | 0.06 |  |  | 15 | 0.61 | 14 | 0.98 | 14 | 1.31 |
| H10N1 | 1 | 0.04 | 1 | 0.06 | 1 | 0.11 |  |  |  |  |  |  |
| H10N2 |  |  |  |  |  |  | 1 | 0.04 | 1 | 0.07 | 1 | 0.09 |
| H10N3 | 1 | 0.04 | 1 | 0.06 | 1 | 0.11 |  |  |  |  |  |  |
| H10N6 | 3 | 0.12 | 2 | 0.12 | 1 | 0.11 | 1 | 0.04 |  |  |  |  |
| H10N7 | 17 | 0.70 | 16 | 0.95 | 9 | 1.03 | 24 | 0.98 | 21 | 1.47 | 13 | 1.22 |
| H10N8 |  |  |  |  |  |  | 1 | 0.04 |  |  |  |  |
| H11N1 |  |  |  |  |  |  | 1 | 0.04 | 1 | 0.07 | 1 | 0.09 |
| H11N2 | 1 | 0.04 | 1 | 0.06 | 1 | 0.11 | 1 | 0.04 | 1 | 0.07 | 1 | 0.09 |
| H11N6 | 1 | 0.04 | 1 | 0.06 | 1 | 0.11 |  |  |  |  |  |  |
| H11N9 | 13 | 0.53 | 12 | 0.71 | 11 | 1.26 | 1 | 0.04 | 1 | 0.07 |  |  |
| H12N5 | 2 | 0.08 | 2 | 0.12 | 1 | 0.11 |  |  |  |  |  |  |
| H12N9 | 1 | 0.04 | 1 | 0.06 | 1 | 0.11 |  |  |  |  |  |  |
